# Reliability Optimization of the Honeycomb Sandwich Structure Based on A Neural Network Surrogate Model

**DOI:** 10.3390/ma16237465

**Published:** 2023-11-30

**Authors:** Zheng Wei, Chunping Zhou, Feng Zhang, Changcong Zhou

**Affiliations:** 1 Key Laboratory for Airborne Hi-Performance Electro-Magnetic Window, RISAC, Ji’nan 250000, China; danielwei1994@163.com (Z.W.); zcp99241@126.com (C.Z.); 2 Department of Engineering Mechanics, Northwestern Polytechnical University, Xi’an 710072, China; changcongzhou@nwpu.edu.cn

**Keywords:** composite structure, neural network, surrogate model, multi-objective optimization, reliability optimization

## Abstract

Composite radomes are usually located in the nose of aircraft and are important structural components that protect radar antenna. The finite element model of a radome structure is developed and analyzed in this article. Single-objective deterministic and reliability optimization models based on the minimum total mass of the radome structure were established, and the layer thickness of each part of the honeycomb sandwich radome structure was considered a design variable. A multi-objective deterministic and reliability optimization model for a radome structure with a minimum total mass and maximum buckling critical load was established, and a particle swarm optimization algorithm was used to solve the problem. Our optimized results satisfied the constraints and utilization rate of materials, and structural safety was improved.

## 1. Introduction

In recent years, because of their high specific stiffness, specific strength, and designability, composite structures have been widely used in various fields [1,2,3]. Radome structures can provide protection for aircraft radar antenna systems in harsh environments and prevent radar system failure owing to lightning strikes, hail, and other environments, as well as serious flight accidents [4,5]. The structural optimization design of composite radome structures, especially the introduction of lightweight and safe designs, has become increasingly important for improving the utilization rate of materials and safety of radome structures with the extensive application of radome structures in aviation, aerospace, and automobile manufacturing [6].

Research on the structural optimization of composite materials, especially composite radomes, is ongoing. The aiming error of radome structures was considered the optimization objective, and the simulated annealing algorithm was adopted by Hsu et al. [7] to optimize radome layup thickness. Optimization results comprehensively improved the structural performance of the radome. A method that combined a genetic algorithm and ray tracing technology was proposed by Meng et al. [8]. The thickness of the radome structure skin and honeycomb was taken as design variables, and the aiming error and power transmission coefficient of the structure were both taken as multi-objectives to optimize the radome structure. The immune cloning algorithm and ray tracing surface integral method were used by Cheng et al. [9] to perform the multi-objective optimization of the aiming error and power transmission coefficient of a radome. Optimization results not only reduced the aiming error but also improved power transmittance. The analytical regularization method was used by Vladimir et al. [10] to perform the numerical optimization of a cylindrical reflector in a radome structure. Xu et al. [11] optimized the aperture error and transmission loss of an airborne radome using a multi-objective particle swarm optimization (PSO) algorithm combined with a three-dimensional (3D) ray tracing method. In this study, a finite element model of the honeycomb sandwich composite radome structure was developed using FORTRAN v95 software, and the parameterization of the finite element model was achieved using a co-simulation of MATLAB v2012b and FORTRAN 95. A back-propagation (BP) neural network surrogate model was established, and a single-target deterministic and reliability optimization model was established with the minimum total mass of the radome structure. Multi-objective deterministic and reliability optimization models were established with minimum total mass and a maximum buckling critical factor of the radome structure. Optimization results were solved using the PSO algorithm, and utilization rates of materials and structural safety were both improved while constraints were satisfied.

## 2. Construction of Radome and Surrogate Models

### 2.1. A Finite Element Model of Radome

**Figure 1:**
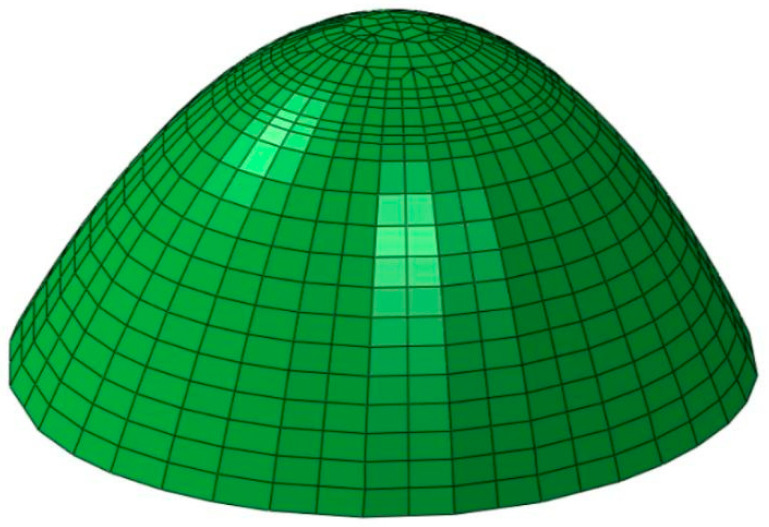
Structural model of the honeycomb sandwich radome.

**Figure 2:**
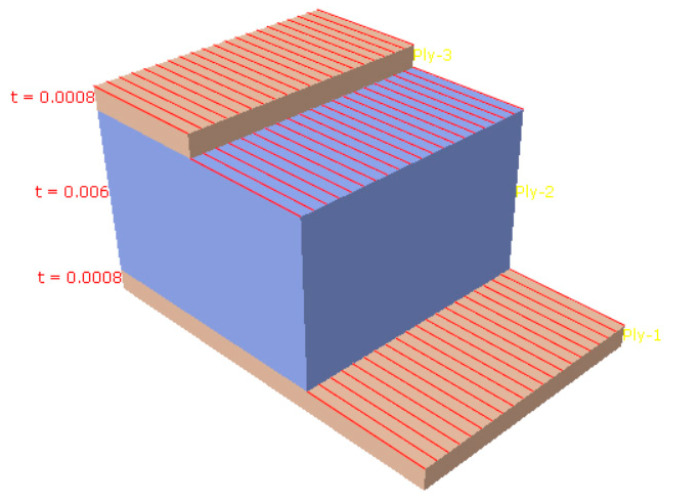
Schematic diagram of a layer.

As an important component of radar systems, radome performance directly affects their function [12]. The honeycomb sandwich radome structure investigated in this study has a strong specific strength, specific stiffness, and a good electromagnetic (EM) wave transmission performance [13], as shown in Figure 1. A schematic diagram of the layer is shown in Figure 2.

**Figure 3:**
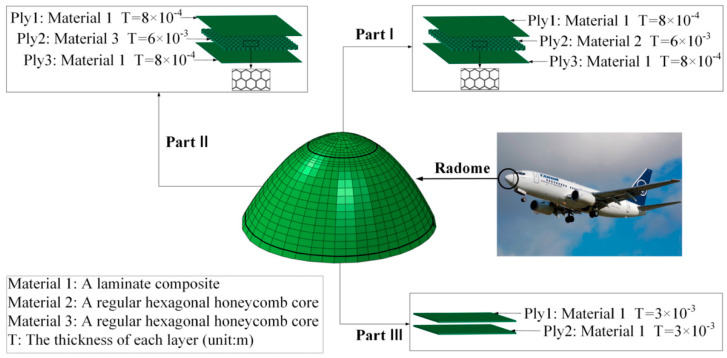
Partition diagram of a radome model.

The structure consists of three materials divided into two honeycomb sandwich materials (materials 2 and 3) and a laminate composite material (material 1). The structure is divided into three parts: Parts I, II, and III. Specifications for each part are as follows:

(1) Part I consists of three layers, the first and third layers are made of material 1 with a thickness of 8 × 10^−4^, and the second layer is made of material 2 with a thickness of 6 × 10^−3^. The angles of the three layers are zero, as shown in the blue part of Figure 3.
(2) Part II consists of three layers: the first and third layers are composed of material 1 with a thickness of 8 × 10^−4^, and the second layer is composed of material 3 with a thickness of 6 × 10^−3^. The angles of the three layers are zero, as shown in the red part of Figure 3.
(3) Part III consists of two layers, both of which are made of material 1 with a thickness of 3 × 10^−3^ and an angle of 0°, as shown in the green part of Figure 3.

The model includes 36 variables and 15 intensity variables. Random variable specifications are shown in Table 1.

### 2.2. Finite Element Analysis

**Figure 4:**
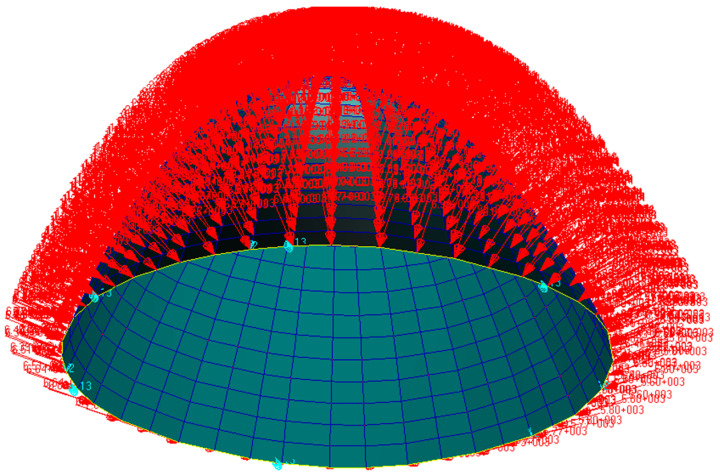
Model loading diagram.

**Figure 5:**
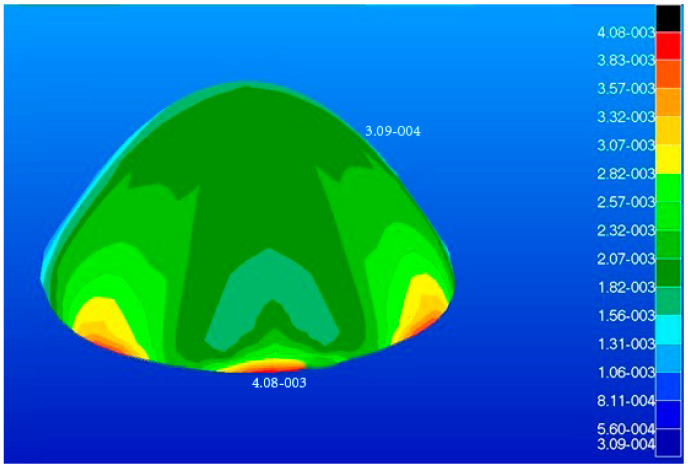
Model displacement diagram (unit: m).

**Figure 6:**
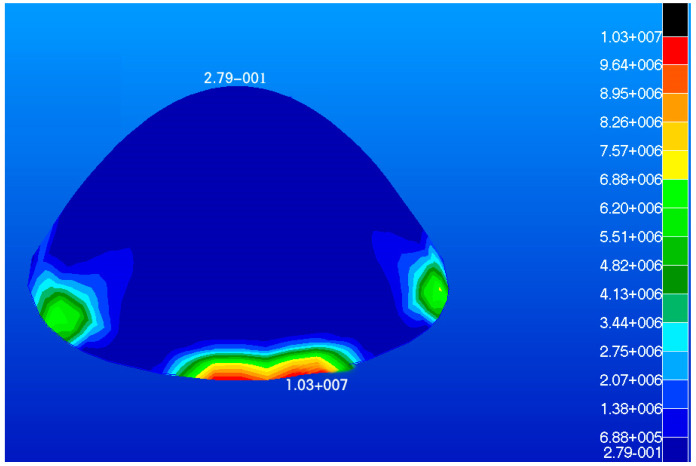
Model stress diagram (Unit: Pa).

**Figure 7:**
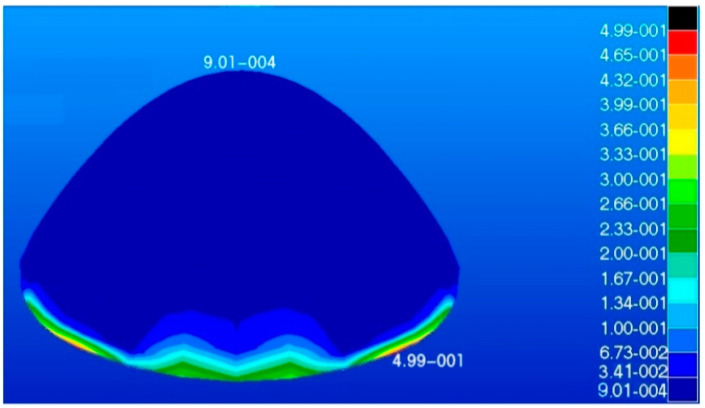
Model strain energy diagram (unit: J).

The radome developed in this study had a dome structure which was modeled using shell elements, where the type of element was S4R and number of elements was 980. The load on the radome is illustrated in Figure 4. The maximum stress, maximum displacement, and total strain energy of the radome structure were calculated and are shown in Figure 5, Figure 6 and Figure 7.

The maximum displacement is 4.08 mm and the maximum response is 10 MPa. The maximum displacement and maximum response occur at the root of the radome. The maximum strain energy is 0.499 J which is located at the root of the radome.

Subfiles in finite element analysis were developed secondarily, and model parameterization was achieved by writing a MATLAB program, which provided data support for the construction of the surrogate model in the following optimization processes.

## 3. Neural Network Surrogate Model

### 3.1. BP Neural Network

**Figure 8:**
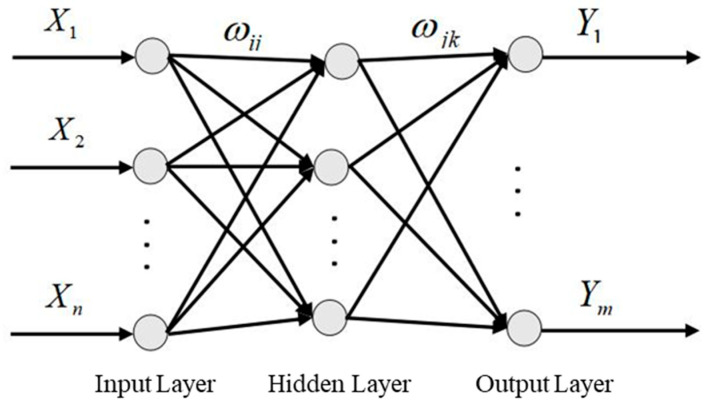
Neural network topology architecture.

The finite element model should be repeatedly calculated during the optimization process of the radome structure. Machine-learning methods, such as neural networks [14,15], support vector machines [16], and Kriging [17], are often used to establish a surrogate model [18,19] of the finite element model to reduce calculation costs. A BP neural network is a multilayer feed-forward neural network, and its main characteristics are forward signal transmission and backward error propagation [20,21]. The topological structure of a BP neural network is illustrated in Figure 8.

In Figure 8, $X_{1},X_{2},\ldots ,X_{n}$ represent the input values of BP neural networks, $Y_{1},Y_{2},\ldots ,Y_{m}$ represent the predicted values of BP neural networks, and $W_{ij}$ and $W_{jk}$ represent the weighted values of BP neural networks. As shown in Figure 8, the BP neural network can be regarded as a nonlinear function. When the number of input nodes is n and the number of output nodes is m, a BP neural network expresses a functional mapping relationship between n independent variables and m dependent variables.

### 3.2. Training Process of the BP Neural Network

**Figure 9:**
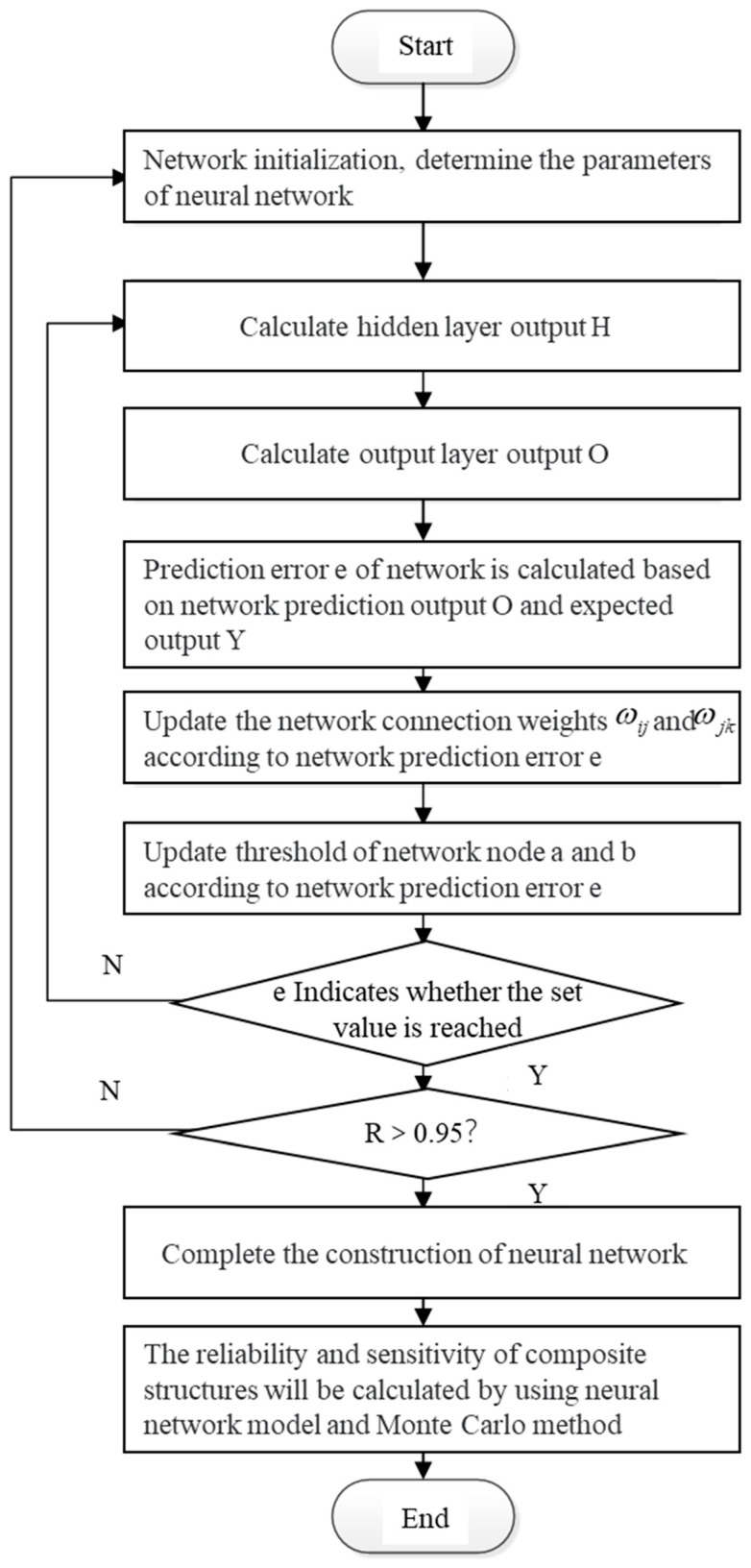
Neural network computing process.

The construction of the surrogate model shown in Figure 9 is based on a BP neural network, as follows:

Step 1: Network initialization. The number of nodes in the input, hidden, and output layers of the network are determined according to the input–output sequence (X, Y) of the system. Connection weights among neurons in the input, hidden, and output layers are initialized, the hidden layer threshold value a and output layer threshold value b are initialized, and the learning rate and neuron excitation function are given.

Step 2: Calculation of the hidden layer output. The hidden layer output *h* is calculated according to the input variable *x*, average connection weights of the input and hidden layers, and the hidden layer threshold value *a*.

(1) $$H_{j}=\int \left(\sum_{i=1}^{n}w_{ij}-\alpha_{j}\right)\;\;\;\;j=1,2,\ldots ,l$$

Here *l* is the number of hidden layer nodes and *f* is the hidden layer excitation function. This function has many forms of expression which we selected in this study

(2) $$f(x)=\frac{1}{1+e^{-x}}$$

Step 3: Output layer output calculation. The connection weight $\omega_{jk}$, threshold *b*, and predicted output *O* of the BP neural network are calculated according to the hidden layer output *H*.

(3) $$O_{k}=\sum_{j=1}^{l}H_{j}w_{jk}-b_{k}\;\;\;\;k=1,2,\ldots ,m$$

Step 4: Error calculation. The network prediction error e is calculated according to the predicted output *O* and expected output y of the network.

(4) $$e_{k}=Y_{k}-O_{k}\;\;\;\;k=1,2,\ldots ,m$$

Step 5: Update the weights. The network connection weight values $\omega_{ij}$ and $\omega_{jk}$ are updated according to the network prediction error.

(5) $$\omega_{ij}=\omega_{ij}+\eta H_{j}(1-H_{j})x(i)\sum_{k=1}^{m}\omega_{jk}-e_{k}\;\;\;\;i=1,2,\ldots ,n;j=1,2,\ldots ,l$$

(6) $$\omega_{jk}=\omega_{jk}+\eta H_{j}e_{k}\;\;\;\;j=1,2,\ldots ,l;k=1,2,\ldots ,m$$

Here η is the learning rate.

Step 6: Threshold update. The network node thresholds *a* and *b* are updated according to the network prediction error *e*.

(7) $$a_{j}=a_{j}+\eta H_{j}(1-H_{j})\sum_{k=1}^{m}\omega_{jk}-e_{k}\;\;\;\;j=1,2,\ldots ,l$$

(8) $$b_{k}=b_{k}+e_{k}\;\;\;\;k=1,2,\ldots ,m$$

Step 7: We determine whether the algorithm iteration is complete. If not, we return to Step 2.

The index for evaluating the training effect of the neural network is the goodness-of-fit *R*, which is expressed as.

(9) $$R=1-\frac{\sum_{i=1}^{n}{\left(y_{i}-{\overset{\wedge }{y}}_{i}\right)}^{2}}{\sum_{i=1}^{n}{\left(y_{i}-{\bar{y}}_{i}\right)}^{2}}$$

If the goodness-of-fit requirement does not meet *R* > 0.95, we return to Step 1 to reset all parameters.

### 3.3. Construction and Accuracy of the BP Neural Network

Three surrogate models were developed in this study, focusing on typical radome structure outputs, such as maximum stress, maximum displacement and total strain energy, and the total mass and buckling critical factor of the structure. Neural network surrogate models were based on the maximum stress, maximum displacement, and total strain energy, named Sur-I, and the neural network surrogate model of the total structural mass, named Sur-II. Sur-III is a neural network surrogate model based on the critical factor of structural buckling. Five hundred sets of input variables were extracted, and 500 sets of output data were calculated using a parameterized finite element model to construct three surrogate models. The 500 samples were divided into three groups, namely, training, verification, and test sets, which accounted for 70%, 15%, and 15%, respectively.

**Figure 10:**
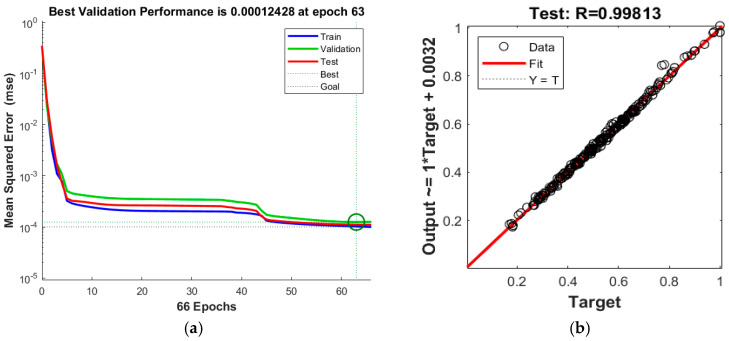
Training effects of the neural network. (**a**) Mean square error of convergence. (**b**) Goodness-of-fit test set.

The neural network of the buckling eigenvalue was constructed, where the number of input layer nodes was 21, representing 21 input variables, the number of hidden layer nodes was 5, and the number of output layer nodes was 1, representing the buckling eigenvalue. After 63 steps of iterative training, the iterative training process of network convergence was terminated when the neural network reached the set error value 10^−4^. The neural network error curve of samples is shown in Figure 10, where the goodness-of-fit of the test set is 0.99813 and neural network accuracy meets requirements.

The training information for surrogate models Sur-II and Sur-III is shown in Table 2, and their respective accuracies satisfy requirements.

## 4. Optimization Method for the Honeycomb Sandwich Structure

### 4.1. Single-Objective Particle Swarm Optimization

**Figure 11:**
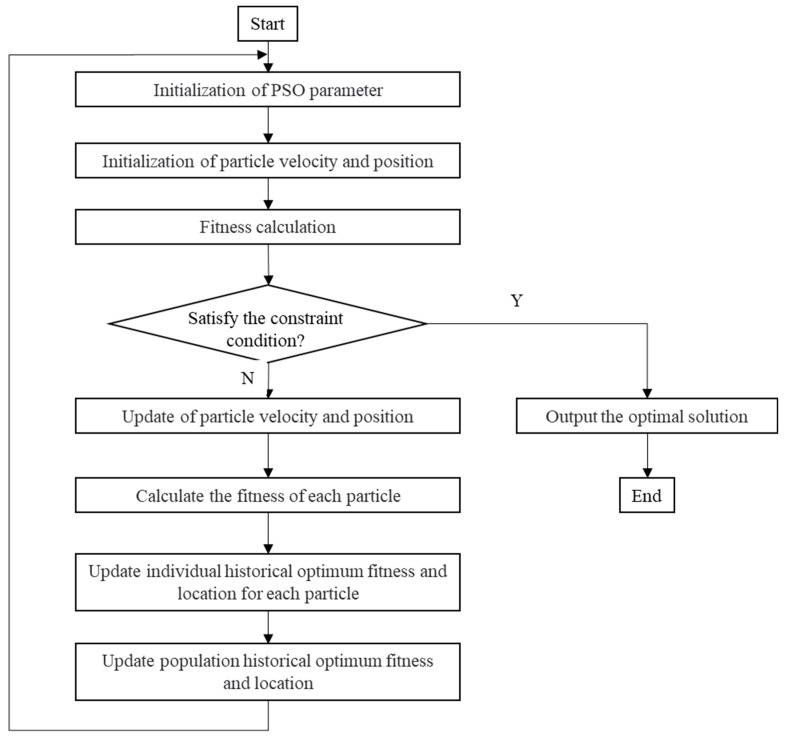
PSO flow diagram.

PSO was proposed by Eberhart et al. in 1995 [22] and is a swarm intelligence algorithm derived from simulations of bird foraging behaviors [23]. After years of continuous development, PSO has become a representative intelligent algorithm [24]. The advantages of PSO are that its algorithms are simple and easy to implement and its optimization ability is strong. The algorithm is widely used in scientific research and engineering applications [25]. PSO algorithms use individuals in a population to collaborate and exchange information. Each individual can be inspired by the experience of its neighbors to determine the search individuals’ speed and direction and ultimately guide the population to the global best. In PSO, each particle in space is assigned an initial random velocity and position. Historical and global optimal particle positions in space are obtained through the mutual exchange of information between particles such that the position of each particle is updated. After multiple particle iterations, particles become clustered around one or more of the best points. The velocity of each particle is expressed by $v_{i}^{}$. The updated formula is as follows:

(10) $$v_{i}^{d+1}=w\times v_{i}^{d}+c_{1}\times r_{1}(pbest-x_{i}^{d})+c_{2}\times r_{2}(gbest-x_{i}^{d})$$

(11) $$x_{i}^{d+1}=x_{i}^{d}+v_{i}^{d+1}$$

where *d* represents the number of iterations; *w* is the inertia weight, which is generally set as a decimal value between 0 and 1; $c_{1}$ is the individual learning factor; and $c_{2}$ is the group learning factor. Generally, $c_{1}$ is equal to $c_{2}$, which ranges from 0 to 4. $r_{1}$ and $r_{1}$ are random numbers between 0 and 1, and they are used to increase the randomness of the search. pbest is the historical optimal position of the particle at iteration d, and gbest is the global optimal position of the particle at iteration d. A PSO flow diagram is shown in Figure 11.

### 4.2. Multi-Objective Particle Swarm Optimization Algorithm

#### 4.2.1. Definition of a Multi-Objective Problem

Multi-objective optimization problems involve optimizing multiple objectives simultaneously [26]. Various goals often interact with each other, and these interactions can be classified as conflicts and harmony [27]. Conflicting relationships mean that as a goal improves, the other worsens, and harmony means that as a goal improves, the other also improves. In multi-objective optimization, the solution to the problem is not unique. In contrast to single-objective optimization, a conflict relationship usually exists between each objective, and it is rare for all objectives to reach the optimal situation simultaneously. Therefore, when solving such problems, we must comprehensively consider all goals and find a solution with a good trade-off between them.

A multi-objective optimization model with m objectives can be defined as follows:

(12) $$\begin{array}{l} MinF(x)=\left(f_{1}\left(x\right),\ldots ,f_{m}\left(x\right)\right)\\ s.t.\left\{\begin{array}{l} x\in \Omega \\ g_{i}(x)\leq 0,i=1,\ldots ,p\\ h_{j}(x)\leq 0,j=1,\ldots ,q \end{array}\right. \end{array}$$

where $x=\left(x_{1},\ldots ,x_{d}\right)\in \Omega$ is a solution composed of d-dimensional decision variables, Ω⊆ℝ is the decision space, $F:\Omega \to {\mathbb{R}}^{m}$ consists of m targets, $g_{i}(x)$ represents the *i*th inequality constraint, and $h_{j}(x)$ represents the jth equality constraint. *p* and *q* represent the numbers of inequality and equality constraints, respectively.

#### 4.2.2. Definition of Pareto Optimal Solution Set

Pareto optimality is the ideal state for resource allocation. The Pareto optimal state refers to a state in which there can be no further Pareto improvement; that is, there is no way of improving some goals without damaging others. At this point, a solution set is required to store a solution that balances the objective function. This is called the Pareto solution set and is known as a non-inferior solution set [28].

The global minimum of the objective function can be defined as

(13) $$\forall x\in \Omega :f\left(x^{*}\right)\leq f\left(x\right)$$

In this case, $f\left(x^{*}\right)$ is the global minimum. Pareto domination is defined as follows:

(14) $$\forall i,j\in \left\{1,2,3,\ldots ,k\right\},\;\;f\left(x_{i}\right)\leq f\left(x_{j}\right)\wedge \exists i,\;j\in \left\{1,2,3,\ldots ,k\right\},\;f\left(x\right)<f$$

The optimal solution is defined as

(15) $$\neg \exists x\in \Omega ,x≺x^{*}$$

The optimal solution set is defined as

(16) $$P=\left\{x^{*}\in \Omega \left|\neg \exists \in \Omega :F(x)≺F(x^{*})\right.\right\}$$

**Figure 12:**
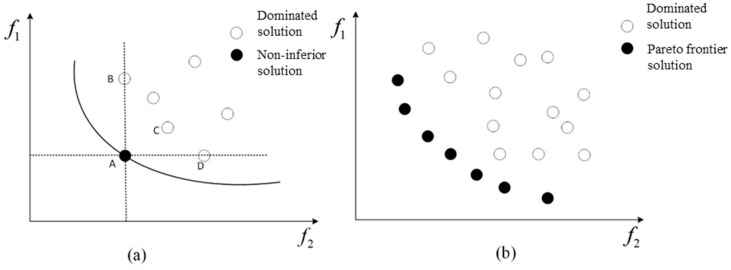
Pareto dominant relationship and frontier solutions. (**a**) Pareto dominant relationship. (**b**) Pareto Frontier Solutions.

The Pareto frontier solution set is defined as

(17) $$PF=\left\{u=F(x)\left|x\in P\right.\right\}$$

where ≺ represents the dominant relationship. For the two solutions $x_{i}$ and $x_{j}$ and the objective function F(x) of multi-objective optimization, if each objective function value $F(x_{i})$ of solution $x_{i}$ is longer than each objective function value $F(x_{j})$ of the solution $x_{j}$, it is called $x_{i}≺x_{j}$. This can be observed from Figure 12a, where A≺B, A≺C, and A≺D. A group of Pareto frontier solutions for the two object spaces is presented in Figure 12b.

#### 4.2.3. Multi-Objective PSO Algorithm

The PSO algorithm, which solves multi-objective optimization problems (MOPSO), was first applied by Coello et al. in 2002. This algorithm has quickly become the main research direction for multi-objective optimization, and has the advantages of being simple, efficient, and a high convergence speed. The update of the particle velocity in the MOPSO optimization problem is improved by updating the particle velocity in PSO. The velocity of the *i*th particle $v_{i}^{d}$ in iteration *d* is defined as follows:

(18) $$v_{i}^{d+1}=w\times v_{i}^{d}+c_{1}\times r_{1}(pbest-x_{i}^{d})+c_{2}\times r_{2}(gbest-x_{i}^{d})$$

**Figure 13:**
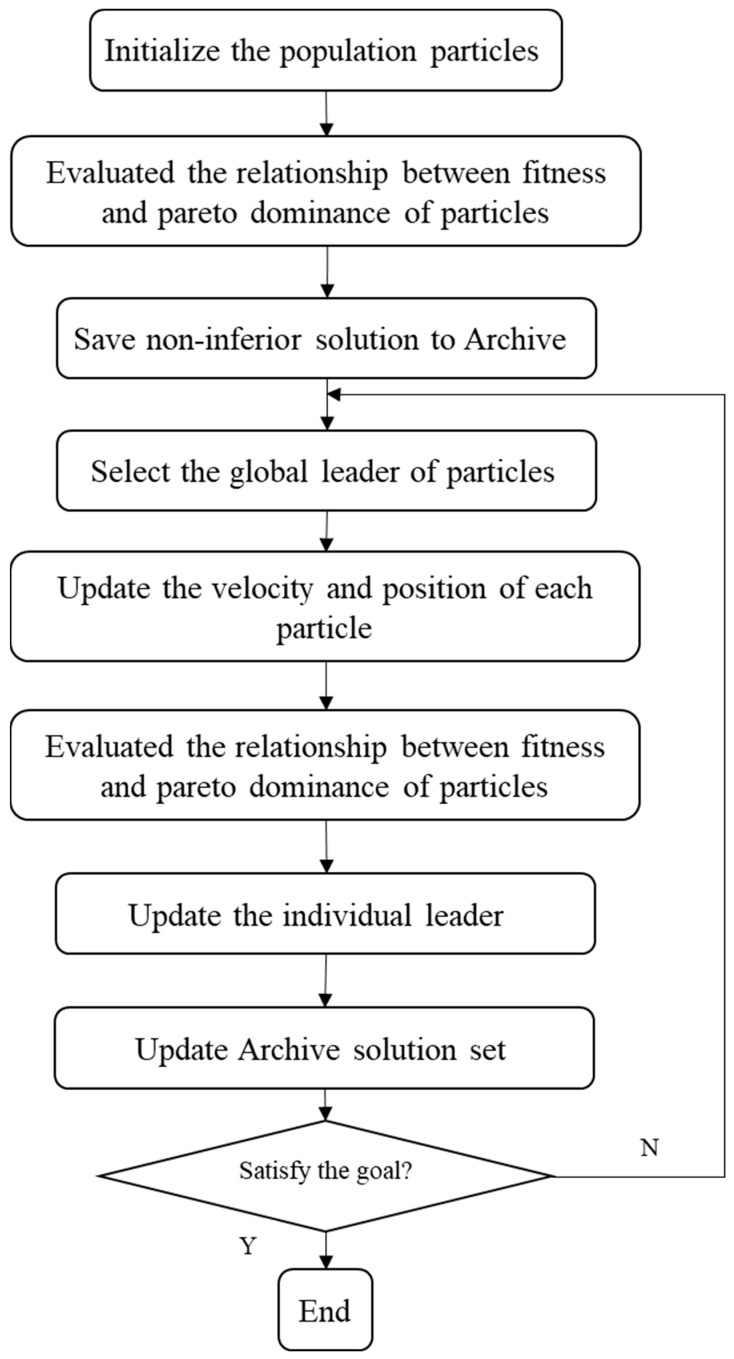
Basic flow of the MOPSO algorithm.

The above equation indicates that the update of $v_{i}^{d}$ in MOPSO is similar to that of single-objective PSO, but the difference lies in the selection of the global optimal solution. In PSO, a globally optimal non-inferior solution $x_{PSO(i)}^{*}$ is generated in each iteration, and a non-inferior solution $x_{PSO(i+1)}^{*}$ is obtained when the algorithm is iterated to the next iteration. The globally optimal non-inferior solution in the next iteration is determined by assessing the non-inferior solution obtained in two iterations. However, in MOPSO, the non-inferior solution $x_{MOPSO(i)}^{*}$ generated each time and the non-inferior solution $x_{MOPSO(i+1)}^{*}$ obtained in the next iteration may not be superior to each other. Therefore, selecting a globally optimal non-inferior solution is impossible in the next iteration based on a simple assessment. Therefore, an external storage, the archive solution set, is required to ensure that the globally optimal non-inferior solution in MOPSO can be found. An archived solution set comprises solutions that cannot be dominated by each other. Figure 13 shows the basic flow of the MOPSO algorithm.

## 5. Optimization Model of the Radome Structure

### 5.1. Design Variables and Constraints

In the optimization of the radome structure, design variables are set as five thickness variables, namely, the thicknesses of Part I Ply1 and Part II Ply1 as denoted by $x_{1}$. The thickness of Part I Ply2 and Part II Ply2 is denoted by $x_{2}$; the thickness of Part I Ply3 and Part II Ply3 is denoted by $x_{3}$; the thickness of Part III ply1 is denoted by $x_{4}$; and the thickness of Part III Ply2 is denoted by $x_{5}$. The maximum stress σ, maximum displacement U, and total strain energy E of the radome structure are considered constraints. [*σ*], [*U*], and [*E*] represent thresholds of σ, U and E. Design variables and constraints are listed in Table 3.

### 5.2. Deterministic Optimization Model

The purpose of the optimal design of an engineering structure is to reduce costs and optimize the performance as much as possible under conditions where the structure meets constraints.

The traditional mathematical model of the deterministic optimal design problem can be expressed as

(19) $$\begin{array}{l} Minf\left(x\right)\\ s.t.\left\{\begin{array}{l} h_{i}(x)\leq 0,i=1,2,\ldots ,m\\ g_{j}(x)\leq 0,j=1,2,\ldots ,n\\ x^{L}\leq x\leq x^{U},x\in R^{n} \end{array}\right. \end{array}$$

where $f\left(x\right)$ represents the objective function; $h_{i}(x)$ and $g_{j}(x)$ represent the inequality and equality constraints, respectively; m and n represent the number of constraints; x represents the design variable; and $x^{L}$ and $x^{U}$ represent the upper and lower limits of the range of the design variable, respectively.

The optimization model for the minimum total mass of the radome structure was established by considering the constraints. The mathematical expression is as follows:

(20) $$\begin{array}{l} MinF=f\left(x_{1},x_{2},x_{3},x_{4},x_{5}\right)\\ s.t.\left\{\begin{array}{l} \sigma_{\max}(x)-\left[\sigma \right]\leq 0\\ U_{\max}(x)-\left[U\right]\leq 0\\ E(x)-\left[E\right]\leq 0 \end{array}\right. \end{array}$$

where $f\left(x_{1},x_{2},x_{3},x_{4},x_{5}\right)$ represents the total mass of the radome structure, and $f\left(x_{1},x_{2},x_{3},x_{4},x_{5}\right)$ represents the five thickness parameters of the radome structure.

The radome structure is directly exposed to external conditions; therefore, its safety must be ensured during operation. The maximum critical buckling load was considered to optimize the structural design and improve radome structure safety. Design variables and constraints in the optimization targeting the maximum critical buckling load were the same as those in the optimization model targeting the minimum total mass of the structure. The mathematical expression for establishing a deterministic multi-objective optimization model with the minimum total mass and maximum critical buckling load of the radome structure is as follows:

(21) $$\begin{array}{l} MinF=\left(f_{1}\left(x\right),f_{2}\left(x\right)\right)\\ s.t.\left\{\begin{array}{l} \sigma_{\max}(x)-\left[\sigma \right]\leq 0\\ U_{\max}(x)-\left[U\right]\leq 0\\ E(x)-\left[E\right]\leq 0\\ x^{L}\leq x\leq x^{U},i=1,2,3,4,5 \end{array}\right. \end{array}$$

where $f_{1}\left(x\right)$ represents the total mass of the radome structure and $f_{2}\left(x\right)$ represents the negative value of the buckling critical load.

### 5.3. Reliability Optimization

The load environment, structural parameters, failure models, design requirements, objective functions, constraints, and design variables are considered deterministic in conventional structural optimization, which simplifies the structural design and calculation process and reduces calculation costs. However, because uncertainty is not considered, the optimization result may not satisfy the constraint conditions when the input variable has a certain fluctuation. Uncertain optimal designs have been proposed to compensate for deficiencies in deterministic optimal designs.

Reliability requirements are incorporated into the constraints of the optimization problem in typical reliability optimization design models, and structural parameters are adjusted to minimize the weight or cost of the structure to satisfy certain reliability requirements of the structural system. The mathematical model is expressed as follows:

(22) $$\begin{array}{l} Minf(x)\\ s.t.\left\{\begin{array}{l} \mathrm{Pr}\left\{g_{i}(X,x)\leq 0\right\}\leq P_{f_{1}}^{*}\;\;i=1,2,\ldots ,m\\ h_{j}(x)\leq 0\;\;(j=1,2,\ldots ,n)\\ g_{k}(x)\leq 0\;\;(k=1,2,\ldots ,p)\\ x^{L}\leq x\leq x^{U},x\in R^{n} \end{array}\right. \end{array}$$

where $\mathrm{Pr}\left\{\cdot \right\}$ is the probability operator, which represents the reliability constraint; x represents the design variable; $g_{i}(X,x)$ represents the *i*th function; and $P_{f_{1}}^{*}$ represents the *i*th reliability objective constraint.

When manufacturing a radome structure, the layup thickness of each part is uncertain owing to technical defects. This paper describes the uncertainty of the five thickness variables using a normal distribution, that is, the design variable $x_{i}∼N(\mu_{i},\sigma_{i}^{2})(i=1,2,3,4,5)$. The maximum stress, maximum displacement, and total strain energy of the radome structure are all reliability constraints that take the minimum mass of the radome structure as the objective function. The mathematical expression for optimization model reliability is as follows:

(23) $$\begin{array}{l} Minf(x)\\ s.t.\left\{\begin{array}{l} \mathrm{Pr}\left\{\sigma_{\max}(x)-\left[\sigma \right]\leq 0\right\}\leq P_{f_{1}}^{*}\\ \mathrm{Pr}\left\{U_{\max}(x)-\left[U\right]\leq 0\right\}\leq P_{f_{2}}^{*}\\ \mathrm{Pr}\left\{E(x)-\left[E\right]\leq 0\right\}\leq P_{f_{3}}^{*}\\ x^{L}\leq x\leq x^{U},x\in R^{n} \end{array}\right. \end{array}$$

where $P_{f_{1}}^{*}$ = $P_{f_{2}}^{*}$ = $P_{f_{3}}^{*}$ = 0.95.

The mathematical expression of the reliability optimization model for the total mass and critical buckling load of the radome structure is as follows:

(24) $$\begin{array}{l} MinF=\left(f_{1}\left(x\right),f_{2}\left(x\right)\right)\\ s.t.\left\{\begin{array}{l} \mathrm{Pr}\left\{\sigma_{\max}(x)-\left[\sigma \right]\leq 0\right\}\leq P_{f_{1}}^{*}\\ \mathrm{Pr}\left\{U_{\max}(x)-\left[U\right]\leq 0\right\}\leq P_{f_{2}}^{*}\\ \mathrm{Pr}\left\{E(x)-\left[E\right]\leq 0\right\}\leq P_{f_{3}}^{*}\\ x^{L}\leq x\leq x^{U},x\in R^{n} \end{array}\right. \end{array}$$

where $P_{f_{1}}^{*}$ = $P_{f_{2}}^{*}$ = $P_{f_{3}}^{*}$ = 0.95.

## 6. Results and Discussion

### 6.1. Deterministic and Reliability Optimization in Single-Objective Optimization Results

**Figure 14:**
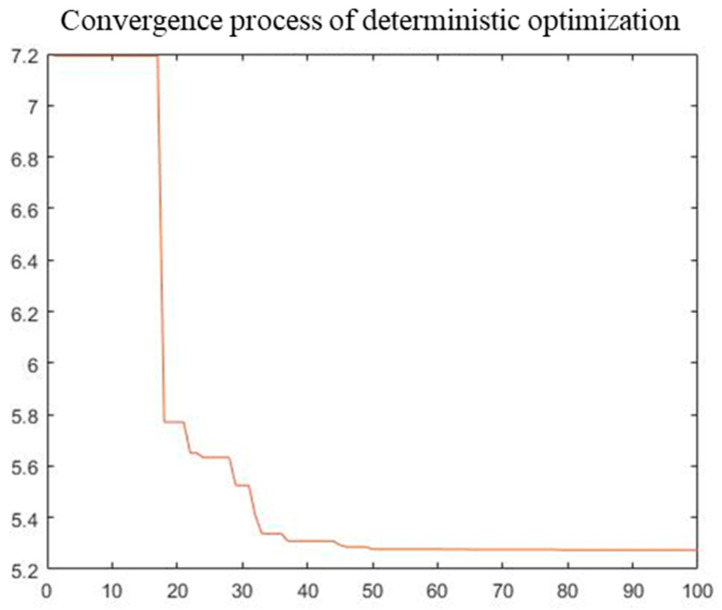
Convergence process of deterministic optimization.

**Figure 15:**
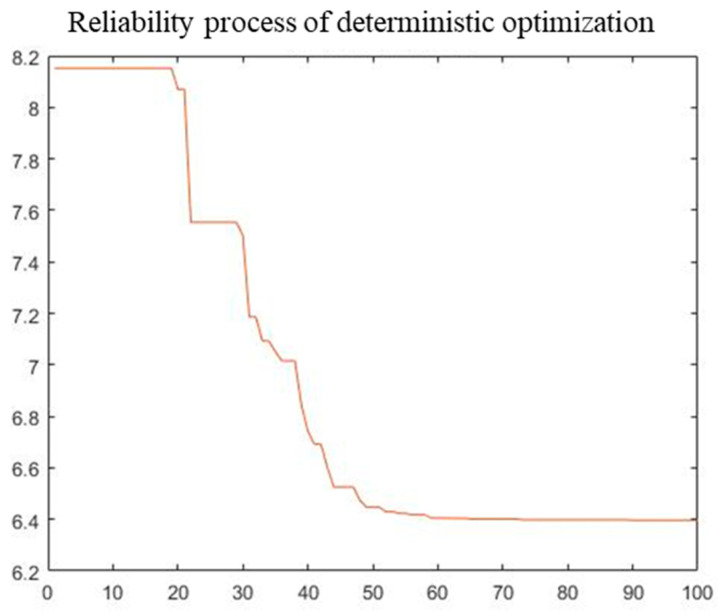
Convergence process of reliability optimization.

A single-objective objective PSO algorithm was used to optimize the total mass of the radome structure. The initial population size was 300. The maximum number of iterations was 100, the inertia weight was set to 0.8, and both self-learning and group learning factors were set to 0.5. Deterministic and reliability results obtained after optimization are listed in Table 4. The PSO convergence process is shown in Figure 14 and Figure 15.

From our optimization results, the five design variables relative to initial values decreased to varying degrees after deterministic and reliability optimization. The total structure mass in deterministic optimization was 5.27, which was reduced by 58.24%. The total structural mass in reliability optimization was 6.40, which was reduced by 49.29%. Reliability optimization results are more conservative than those for deterministic optimization owing to the addition of reliability constraints. The convergence process of optimization indicates that the convergence speed of deterministic optimization is faster, and fitness remains unchanged after approximately the 50th iteration. However, the convergence speed of reliability optimization is relatively slow, and fitness remains unchanged after approximately the 60th iteration. The difference in convergence speed between the two is mainly because the reliability calculation reduces the convergence speed of the reliability optimization.

### 6.2. Deterministic and Reliability Optimization in Multi-Objective Optimization Results

**Figure 16:**
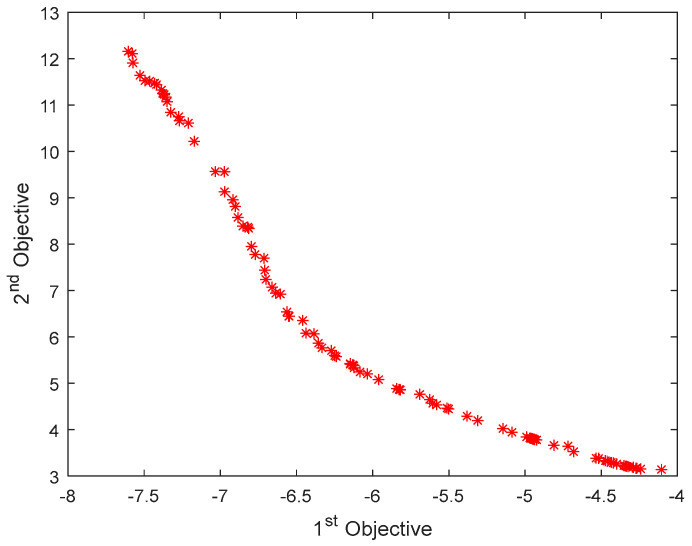
Pareto frontier of deterministic optimization.

**Figure 17:**
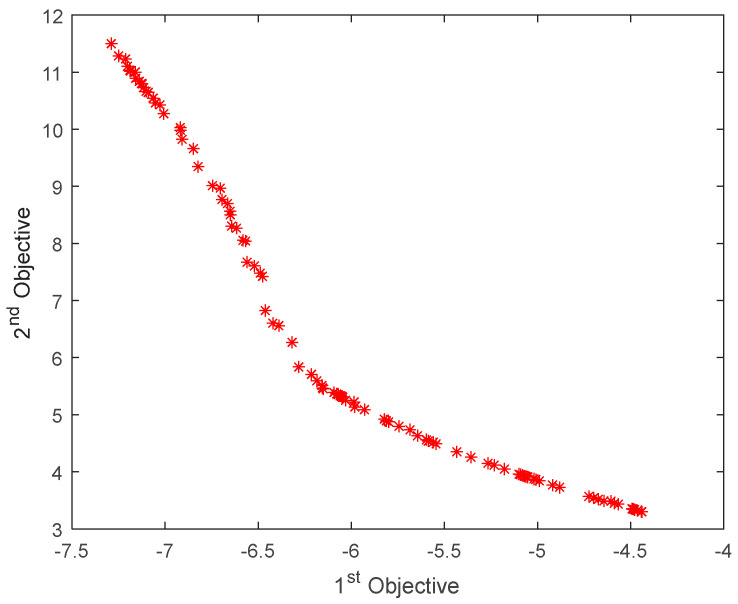
Pareto frontier of reliability optimization.

A multi-objective PSO algorithm was used to optimize the total mass and buckling critical load of the radome structure with an initial population of 200. The maximum number of iterations was set to 100, and the upper limit for external storage (archive) disaggregation was set to 100. The inertia weight was 0.5, the individual learning factor and the population learning factor were set to 1 and 2, respectively, and 100 groups of feasible solutions were obtained after optimization. Of these, 10 groups of data were randomly selected, as shown in Table 5. The Pareto frontier solution set obtained by multi-objective optimization is shown in Figure 16 and Figure 17.

According to multi-objective optimization results, the critical buckling load of the structure increases and the weight of the structure decreases after deterministic and reliability optimization. When selecting the most satisfactory solution in the non-inferior solution set, response weights can be set for each objective function according to designer preferences. Subsequently, multi-objective optimization can be weighted and transformed into a single-objective problem that directly compares the size for processing. A comparison of Pareto frontiers obtained via deterministic and reliability optimization indicates that the critical load and total mass obtained via reliability optimization are more conservative than those obtained via deterministic optimization, indicating that some material properties are sacrificed. However, the safety of the structure can be improved when the uncertainty of the layer thickness of each part is considered during the radome structure design process.

## 7. Conclusions

(1) A finite element model of the radome structure was established, and finite element analysis performed based on maximum stress, maximum displacement, and total strain energy. The model was parameterized using MATLAB and FORTRAN co-simulations.
(2) In deterministic optimization, the total mass of the radome structure decreased, and the material utilization rate increased, whereas optimization results satisfied constraints. The critical buckling force of the radome increased, and the safety of the radome structure was improved.
(3) The uncertainty of parameters was considered in the reliability optimization. The total mass of the radome structure decreased, and the material utilization rate increased, whereas optimization results satisfied reliability constraints. In addition, structural safety was improved with an increase in critical buckling force.
(4) The research results of this paper can be applied to the optimal design of aircraft radome structures considering uncertainty, but the uncertainty generated by product manufacturing processes is not considered in this paper, and the influence of manufacturing parameter uncertainty on structural strength should be focused on in future research.

## Figures and Tables

**Table 1:** Random variable information.

Number	Variable Name	Variable	Mean	Standard Deviation	Distribution Pattern
1	Mat1E11	The modulus in 11 direction of material 1	1.55 × 10^10^ Pa	1.55 × 10^9^ Pa	Normal
2	Mat1E22	The modulus in 22 direction of material 1	1.55 × 10^10^ Pa	1.55 × 10^9^ Pa	Normal
3	Mat1G12	The modulus in 12 direction of material 1	7.3 × 10^9^ Pa	7.3 × 10^8^ Pa	Normal
4	Mat1G13	The modulus in 13 direction of material 1	3.6 × 10^9^ Pa	3.6 × 10^8^ Pa	Normal
5	Mat1G23	The modulus in 23 direction of material 1	3.6 × 10^9^ Pa	3.6 × 10^8^ Pa	Normal
6	Mat1Ro	Density of material 1	1828 kg/m^3^	182.8 kg/m^3^	Normal
7	Mat2E11	The modulus in 11 direction of material 2	4.5 × 10^4^ Pa	4.5 × 10^3^ Pa	Normal
8	Mat2E22	The modulus in 22 direction of material 2	4.5 × 10^4^ Pa	4.5 × 10^3^ Pa	Normal
9	Mat2G12	The modulus in 12 direction of material 2	2.1 × 10^4^ Pa	2.1 × 10^3^ Pa	Normal
10	Mat2G13	The modulus in 13 direction of material 2	3.83 × 10^7^ Pa	3.83 × 10^6^ Pa	Normal
11	Mat2G23	The modulus in 23 direction of material 2	1.87 × 10^7^ Pa	1.87 × 10^6^ Pa	Normal
12	Mat2Ro	Density of material 2	65 kg/m^3^	6.5 kg/m^3^	Normal
13	Mat3E11	The modulus in 11 direction of material 3	4.5 × 10^6^ Pa	4.5 × 10^5^ Pa	Normal
14	Mat3E22	The modulus in 22 direction of material 3	4.5 × 10^6^ Pa	4.5 × 10^5^ Pa	Normal
15	Mat3G12	The modulus in 12 direction of material 3	4.5 × 10^6^ Pa	4.5 × 10^5^ Pa	Normal
16	Mat3G13	The modulus in 13 direction of material 3	1.5 × 10^7^ Pa	1.5 × 10^6^ Pa	Normal
17	Mat3G23	The modulus in 23 direction of material 3	2.53 × 10^7^ Pa	2.53 × 10^6^ Pa	Normal
18	Mat3Rou	Density of material 3	65 kg/m^3^	6.5 kg/m^3^	Normal
19	M1	Skin thickness 1	8 × 10^−4^ m	8 × 10^−5^ m	Normal
20	M2	Thickness of honeycomb sandwich	6 × 10^−3^ m	6 × 10^−4^ m	Normal
21	M3	Skin thickness 2	3 × 10^−3^ m	3 × 10^−4^ m	Normal

**Table 2:** Training information of surrogate models.

Surrogate Models	*n*	*p*	m	Error	Iteration	R
Total mass	5	5	1	1 × 10^−4^	108	0.99946
Critical factor of buckling	5	9	1	1 × 10^−4^	44	0.99706

**Table 3:** Design variables and constraint information.

	Design Variables/m					Constraints		
	$x_{1}$	$x_{2}$	$x_{3}$	$x_{4}$	$x_{5}$	[*σ*]/Pa	[*U*]/m	[*E*]/J
Lower limit	0.0001	0.001	0.0001	0.0005	0.0005	32,000,000	0.007	20
Upper limit	0.0015	0.01	0.0015	0.006	0.006			

**Table 4:** Single-objective optimization results.

Process of Optimization	Mode of Optimization	Design Variables					Objective Function
		$x_{1}$	$x_{2}$	$x_{3}$	$x_{4}$	$x_{5}$	$f\left(x\right)$
	Initial value	0.0008	0.006	0.0008	0.003	0.003	12.62
After optimization	Deterministic	0.0001	0.0001	0.00028	0.001	0.00233	5.27
	Reliability	0.0001	0.001	0.0004	0.001	0.00234	6.40

**Table 5:** Multi-objective optimization results.

Process of Optimization	Mode of Optimization	Design Variables					Objective Function	
		$x_{1}$	$x_{2}$	$x_{3}$	$x_{4}$	$x_{5}$	$f_{1}\left(x\right)$	$f_{2}\left(x\right)$
	Initial value	0.0008	0.006	0.0008	0.003	0.003	−4.9794	12.62
After optimization	Deterministic	0.00048	0.00998	0.00043	0.006	0.00567	−7.41818	11.44283
		0.00054	0.00999	0.00038	0.006	0.00525	−7.20989	10.61179
		0.00052	0.00999	0.00043	0.006	0.00368	−7.03305	9.566278
		0.00056	0.00998	0.00046	0.006	0.00150	−6.77129	7.775304
		0.00054	0.00999	0.0004	0.006	0.00510	−7.26849	10.6594
		0.00049	0.00998	0.0005	0.006	0.00188	−6.81962	8.368969
		0.00055	0.01	0.0004	0.006	0.00332	−6.97217	9.131097
		0.00050	0.01	0.00016	0.006	0.0005	−4.95449	3.8081
		0.00061	0.00999	0.00021	0.006	0.0005	−5.50226	4.44527
		0.00066	0.00998	0.00042	0.006	0.0005	−6.55072	6.44738
	Reliability	0.00074	0.01	0.00018	0.006	0.0005	−5.4336	4.349
		0.00047	0.01	0.00039	0.006	0.00193	−6.4769	7.42226
		0.00056	0.01	0.00036	0.006	0.00124	−6.3889	6.55908
		0.00048	0.01	0.00036	0.006	0.00568	−7.1381	10.8395
		0.0005	0.01	0.00038	0.006	0.00225	−6.5596	7.67189
		0.0005	0.01	0.00036	0.006	0.0035	−6.6648	8.69748
		0.00067	0.01	0.0001	0.006	0.0005	−4.6055	3.48521
		0.00046	0.01	0.00039	0.006	0.0044	−6.9086	9.82462
		0.00046	0.01	0.00037	0.006	0.00589	−7.202	11.1023
		0.00063	0.01	0.00029	0.006	0.0005	−6.0303	5.25114

## Data Availability

Data are contained within the article.
